# Comparing CD8+ T cell ENV epitopes of two HLA class I alleles associated with different outcomes of HIV-1 infection with broadly neutralizing antibodies epitope targets discovered to date

**DOI:** 10.3389/fimmu.2026.1786452

**Published:** 2026-04-13

**Authors:** Akhil Ramdoyal, Christina A. Daniuk, Robert Were Omange, Joshua Kimani, Ma Luo

**Affiliations:** 1 Max Rady College of Medicine, University of Manitoba, Winnipeg, MB, Canada; 2 National Microbiology Laboratory Branch, Public Health Agency of Canada, Winnipeg, MB, Canada; 3 Biomarker Sciences, Gilead Sciences, Foster City, CA, United States; 4 Department of Medical Microbiology and Infectious Diseases, University of Manitoba, Winnipeg, MB, Canada; 5 Department of Medical Microbiology, University of Nairobi, Nairobi, Kenya

**Keywords:** broadly neutralizing antibodies (bNab), CD8+ T cells, HIV-1 envelope (ENV), HLA-A*01:01, HLA-B*07:02, natural immunity, Pumwani cohort, immunotherapy

## Abstract

The HIV vaccine field has swung between humoral-based to cell-mediated approaches; however the consensus has evolved toward the understanding that an induction of both may be important for optimal protection. HLA class I molecules present viral epitopes to initiate CD8+ T cell responses to HIV-infected cells while broadly neutralizing antibodies (bNAbs) primarily target free virions. In this study, we systematically compared HIV-1 ENV CD8+ T cell epitopes restricted by two HLA class I alleles associated with divergent HIV-1 infection outcomes with bNAb epitopes reported to date in the literature. To our knowledge, this represents one of the first attempts of examining and reconciling the targets of cellular and humoral immunity in the context of differing HIV infection outcomes. Among 1820 peptides overlapping HIV-1 clades A and D ENV, A*01:01 (associated with slower HIV-1 acquisition) bound to 20, while B*07:02 (associated with susceptibility) bound to 64. No significant differences in affinity or off-rate were noted between A*01:01 and B*07:02 epitopes, and a higher proportion of A*01:01 epitopes are located within constant ENV regions than B*07:02 epitopes (C:V ratio 3:1 versus 2:1, respectively). Of the epitopes and variants discovered in this study, 7 of 20 (35%) A*01:01 and 11 of 64 (17%) B*07:02 binders overlapped with known bNAb target sites. All CD8+ T cell epitopes overlapping CD4-binding site clusters were also bNAbs targets. Notably, all A*01:01 T cell epitopes overlapping bNAb epitopes were within conserved ENV regions, and all but one involved glycan-dependent bNAb binding. The epitope landscape analysis therefore showed the allele associated with slower HIV-1 infection rates presents a narrower repertoire of ENV CD8^+^ T cell epitopes, that preferentially overlaps bNAb target sites, consistent with coordinated immune targeting between cellular and humoral arms of immunity.

## 1 Introduction

The HIV/AIDS pandemic continues to pose a major global health challenge. Current estimates indicate that approximately 40 million people are living with HIV worldwide, and despite the transformative impact of antiretroviral therapy (ART) on survival and transmission, more than 1.3 million new infections were reported in 2023 alone (1). More than four decades after the identification of HIV as the AIDS-causing agent, the development of an effective prophylactic vaccine remains elusive (2–6). The extraordinary genetic diversity and rapid mutational capacity of the virus, its preferential targeting of CD4^+^ T cells (key coordinators of adaptive immunity) and the early establishment of viral reservoirs through integration into the host genome shortly after infection are often cited as challenges that past vaccine candidates failed to overcome (7, 8). Early HIV vaccine strategies largely emphasized either humoral or cell-mediated immunity in isolation. However, mounting evidence now supports the view that durable protection against HIV will likely require the coordinated engagement of both arms of the adaptive immune response (2). CD8^+^ T cells play a central role in recognizing and eliminating HIV-infected cells after presentation of viral peptides by HLA class I molecules of viral infected cells. In parallel, broadly neutralizing antibodies (bNAbs) can fight infection by binding to antigenic determinants on viral surface proteins, neutralizing free virions and preventing cellular entry. BNAbs induction is multifactorial, with key determinants including viral load, infection duration, viral diversity, and host factors such as HLA genotype and ethnicity (9, 10). BNAbs can interfere or neutralize the first few rounds of viral replication, thereby giving time for the cellular immune response to activate. Nonetheless, bNAbs typically arise only after prolonged antigen exposure and extensive somatic hypermutation, underscoring the need to better understand how humoral and cellular immunity may intersect during natural HIV infection.

Unique insights into protective immune responses against HIV have emerged from the Pumwani sex worker cohort in Nairobi, Kenya (11–15). Established in 1985 during the early years of the HIV epidemic, this longitudinal cohort includes a subset of women who remained HIV-seronegative despite repeated high-risk exposure during a period preceding the availability of ART. Importantly, this persistent seronegativity cannot be explained by differences in exposure risk or undetected infection, suggesting the presence of intrinsic protective immune mechanisms. As such, the Pumwani cohort represents a rare opportunity for studying natural resistance to HIV.

Previous studies have demonstrated the implication of specific human leukocyte antigen (HLA) class I and class II alleles within the Pumwani cohort, as well as distinct HIV-specific CD8^+^ and CD4^+^ T cell responses (7, 11–13). Among these, HLA class I molecules, which are incredibly diverse, are central to the presentation of endogenously derived viral peptides to CD8^+^ T lymphocytes that then mediate the elimination of infected host cells (14, 15). Accordingly, the HLA class I pathway is the primary focus of the present study. During viral infection, intracellular viral proteins are processed by the proteasome into short peptide fragments that are subsequently loaded onto HLA class I molecules and transported to the surface of nucleated cells as peptide–MHC complexes. These complexes are then surveilled by CD8^+^ T cells. Peptide binding to individual HLA class I alleles is governed by a limited number of anchor residues - typically two or three positions within the peptide sequence - that conform to allele-specific binding motifs and confer stability within the peptide-binding groove (16).

Within the Pumwani sex worker cohort, prior genetic association analyses have identified HLA-A*01:01 as significantly enriched among women who remain HIV-1 seronegative despite repeated exposure (p = 0.016; odds ratio = 1.7; 95% confidence interval [CI]: 1.1–2.7). In contrast, HLA-B*07:02 has been found at higher frequency among HIV-1–infected individuals (p = 0.035; odds ratio = 0.38; 95% CI: 0.14–2.1) and has also been associated with elevated viral loads and accelerated disease progression (7). Based on these different HIV infection outcomes, A*01:01 and B*07:02 were selected as the alleles of interest for this investigation. Since HLA class I molecules restrict which viral epitopes are presented to CD8+ T cells, defining allele-specific epitope repertoires not only provides a rational approach to understanding how cellular immunity contributes to protection or susceptibility, but also provides clues as to how bNAbs may interact with these epitopes, if at all. The HIV-1 envelope glycoprotein (ENV) has a central role in viral entry and is the primary target of bNAbs (17–20). ENV occupies a unique immunological position: it is the dominant antigenic target of neutralizing antibodies while also containing CD8^+^ T cell epitopes presented on infected cells (19–24). Whether and how ENV-specific cellular and humoral responses intersect—particularly in individuals with protective versus susceptible HLA alleles—remains poorly understood. To our knowledge, this study represents one of the first attempts of systematically examining the ENV epitope landscape with the goal of reconciling the targets of cellular and humoral arms of immunity in the context of differing HIV-1 infection outcomes.

## 2 Materials and methods

### 2.1 Study cohort and ethics statement

The participants who took part in this study were women enrolled in the open and longitudinal Pumwani Sex Worker cohort established in 1985 in Nairobi, Kenya to study sexually transmitted diseases (25). All enrollees have been followed biannually after enrollment and a subgroup of them remain serologically and PCR negative for HIV despite repeated exposure through high-risk sex work (11). The ethical approval for the study was obtained from the Institutional Review Boards at the Universities of Manitoba and Nairobi. All study participants gave informed written consent to participate.

### 2.2 Peptide synthesis, epitope screen and confirmation

A comprehensive library of overlapping peptides covering the HIV-1 envelope (ENV) protein of clade A consensus and clade D consensus were synthesized by JPT Peptide Technologies (Berlin, Germany). The 2024 consensus ENV sequences of HIV subtypes A1, A2 and D were generated from HIV database (http://www.hiv.lanl.gov/content/sequence/NEWALIGN/align.html) and downloaded. The A1, A2 and D ENV consensus sequences were aligned with MEGA 5.2 (26) and the sequence variations were identified. These clades were chosen to reflect the exposure context of the cohort as studies of women in Nairobi show that Clade A accounts for the majority of infections (typically around 70–90%), while Clade D represents approximately 10–20% of cases (27–29). In total, 1820 9-mer sequences overlapping by eight amino acids incorporated consensus sequence variants to account for regional viral diversity.

Peptide binding was assessed using the iTopia Epitope Discovery System (30) with A*01:01 and B*07:02 kits purchased from Beckman Coulter (San Diego, CA). The iTopia system consists of peptide binding, dissociation (off-rate), and affinity assays while and the iTopia kits include the monomer-coated plates, assay buffers, anti-HLA-ABC-FITC conjugated monoclonal antibody, β2M and a positive control peptide. The anti-HLA antibody binds only to a properly folded peptide-HLA Class 1 complex. The synthetic 9-mer peptides were incubated with biotinylated soluble HLA class I monomers in 96-well microtiter plates under optimal conditions (11 μM peptide incubated with β2M, anti-MHC mAb and plate bound MHC heavy chain at 21 °C for 18 h) (30) using a fluorimeter (Spectra Max Gemini from Molecular Devices). Results of the peptide binding assay were imported into the iTopia software and expressed as a percent of the positive control provided in each kit. Peptides demonstrating binding activity exceeding 30% of the fluorescence generated by the positive control were classified as binders and selected for further analysis of binding affinity and off-rate (t_1/2_). Affinity assays were performed across peptide concentrations ranging from 10^-4^ to 10^-9^ M, with half-maximal effective concentration (ED_50_) values defined as the peptide concentration achieving 50% of maximal MHC binding, corresponding to the midpoint of the sigmoidal binding curve (7, 30–32). Peptide–MHC stability was evaluated using off-rate assays, which yield a t_1/2_ value or the time required for 50% of the bound peptide to dissociate along the exponential decay curve. Fluorometric readings were taken at each time point after washing the binding peptides that have been incubated on each allele-specific plate. Data were analyzed using the iTopia software and GraphPad Prism version 9.5. Group differences were determined using the Mann-Whitney U test.

To verify that HLA-binding peptides identified with the iTopia Epitope Discovery System were true T cell epitopes and capable of eliciting antigen-specific cellular immune responses, interferon-γ (IFN-γ) enzyme-linked immunospot (ELISpot) assays using fresh peripheral blood mononuclear cells (PBMCs) from HLA-typed study participants were performed as previously described (7, 30, 32). Peptides (9 amino acid) identified by the iTopia epitope discovery system were synthesized (Sigma Genosys, Oakville, ON). The peptide stocks were dissolved in dimethyl sulfoxide (DMSO), and the stocks were diluted to a final concentration of 10 mg/ml in RPMI medium for ELISpot assays. Ninety-six well nitrocellulose plates were coated with anti-IFN-γ monoclonal antibody (mAb; Mabtech, Nacka Strand, Sweden) followed by blocking with R-10 media. PBMCs were suspended in RPMI media, and 1 × 10^5^ cells per well were stimulated in duplicate overnight at 37°C (with CO2) with individual peptides at a concentration of 2.5 µg/mL, without pooling, 1 mg/ml phytohemagglutinin (PHA, as positive control) or media (background). After incubation, the cells were discarded, plates were washed and incubated with a biotinylated anti-IFNγ mAb (Mabtech) followed by streptavidin-conjugated alkaline phosphatase (Mabtech). Plates were developed using an alkaline phosphatase-conjugate substrate kit (Bio-Rad Labora-tories, Ontario, Canada) and the spot-forming units (SFUs) were counted using an automated ELISPOT reader (Autoimmun Diagnostika GmbH, Strassberg, Germany). Responses were considered positive when peptide stimulation resulted in at least 50 SFU per million PBMCs following background subtraction, provided that the positive control was successful. Donors were heterozygous for either A*01:01 or B*07:02 (**Table 1**). A total of ten donors per allele were selected to provide sufficient power to detect ≥1.5-fold differences in IFN-γ responses, consistent with prior epitope-mapping studies. Donor-level summaries were compared between allele groups using the Mann–Whitney U test, with analyses additionally stratified by HIV serostatus where indicated.

**Table 1:** Cohort data and HLA class I alleles of all patients tested in ELISpot assays using fresh PBMC.

Allele	Patient #	HIV status at cohort entry	HIV status at sampling time	Assessment phenotype	PBMC sampling and ELISpot Assay time	Enrolment date	Last day	Days of follow-up
A*01:01	1938	Neg	Neg	HESN	May, 2010	27-Feb-2001	08-May-2010	3311
	2178	Pos	Pos	ND	May, 2010	11-Jun-2003	08-May-2010	2487
	2261	Pos	Pos	ND	May, 2010	05-Aug-2004	08-May-2010	2073
	2297	Pos	Pos	ND	May, 2010	24-Oct-2005	02-Dec-2010	1838
	2326	Pos	Pos	ND	May, 2010	27-Mar-2006	01-Feb-2011	1744
	2426	Pos	Pos	ND	May, 2010	14-Sep-2006	02-Dec-2010	1518
	2463	Neg	Neg	Seroconverter	May, 2010	27-Sep-2006	24-Jan-2011	1557
	2468	Pos	Pos	ND	May, 2010	28-Sep-2006	19-Nov-2010	1491
	2646	Pos	Pos	ND	May, 2010	20-Feb-2007	31-Jan-2011	1421
	2672	Pos	Pos	ND	May, 2010	28-Feb-2007	20-Jan-2011	1400
	2231	Neg	Pos	Seroconverter	May, 2010	08-Sep-2003	08-May-2010	2400
B*07:02	2320	Neg	Neg	NEG	May, 2010	22-Mar-2006	29-Jun-2011	1897
	2337	Neg	Neg	NEG	May, 2010	10-Apr-2006	08-May-2010	1468
	2400	Pos	Pos	ND	May, 2010	14-Aug-2006	08-May-2010	1344
	2423	Neg	Neg	NEG	May, 2010	13-Sep-2006	19-Jan-2011	1566
	2425	Neg	Neg	NEG	May, 2010	14-Sep-2006	10-Jun-2011	1706
	2427	Pos	Pos	ND	May, 2010	14-Sep-2006	19-Nov-2010	1505
	2545	Neg	Neg	NEG	May, 2010	30-Oct-2006	28-Feb-2011	1558
	2619	Pos	Pos	ND	May, 2010	05-Feb-2007	25-Jan-2011	1430
	2644	Neg	Neg	NEG	May, 2010	19-Feb-2007	19-Jan-2011	1410

HESN, HIV-Exposed PCR Seronegative; ND, non-defined. Estimated seroconversion date for patient 2463 was 15-Oct-2010, and 22-Mar-2005 for patient 2231. HIV status was determined using serologic testing and PCR.

### 2.3 Identification of broadly neutralizing antibodies targets from literature

Broadly neutralizing antibodies (bNAbs) and their corresponding HIV-1 ENV target regions were compiled through a comprehensive literature review intended to capture bNAbs reported to date, independent of whether their epitopes were expected to overlap HLA class I–restricted epitopes identified in this study. Searches were conducted primarily in PubMed, supplemented by targeted resources commonly used for HIV epitope annotation (including the Los Alamos National Laboratory (LANL) HIV Immunology/Sequence Database, where applicable). Search terms included identifiers for HIV-1 broadly neutralizing antibodies (e.g., “broadly neutralizing antibody”, “antibodies”, “bNAbs”, “HIV-1”) and site-based terms (e.g., “CD4 binding site”, “V1/V2 apex”, “V3 glycan”, “high-mannose patch”, “fusion peptide”, “gp120–gp41 interface”, “MPER”, “neutralization breadth”).

No restrictions were applied with respect to publication date, viral clade, or experimental mapping approach. BNAbs were eligible for inclusion if the literature provided sufficient information to define their ENV target region in a manner that could be mapped onto a reference ENV sequence: either the exact position is known, or a position range within an error margin of 3 amino acids of the HIV-1 sequence could be inferred with high confidence. Epitope definitions were extracted preferentially from original discovery papers and/or subsequent high-resolution epitope characterization studies, including those using neutralization panels, site-directed mutagenesis or glycan knockouts, neutralization escape profiling, competition/binding assays, and where available, X-ray crystallography or cryo-electron microscopy of bNAb–Env complexes (10, 33–54).

### 2.4 Mapping A*01:01, B*07:02 and bNAbs epitopes to the HIV-1 Clade A/D sequence consensus

The T cell epitopes and epitope variants of A*01:01 and B*07:02 were mapped onto the HIV-1 clade A/D ENV consensus sequence (LANL HIV Sequence Database). The reported bNAb positions were also mapped onto the same consensus ENV sequence. When multiple binding sites existed for a given bNAb, we included all validated sites. Mapped bNAb epitopes on the consensus ENV were double-checked to preserve correct positioning (for example, relative to known ENV landmarks such as variable loops or highly conserved motifs) before proceeding to overlap analysis. An “overlap” was defined as an intersection of at least 1 ENV amino acid at a position also occupied by an amino acid of a 9-mer T cell epitope and a bNAb epitope.

Furthermore, we used the ChimeraX software (v.2025-05-21) together with the HIV-1 cryo-EM model PDB6PWU (54) to map the epitopes discovered in this study with the ENV trimer’s higher order structure.

## 3 Results

### 3.1 A*01:01 and B*07:02 were associated with different outcomes of HIV infection

**Figure 1:**
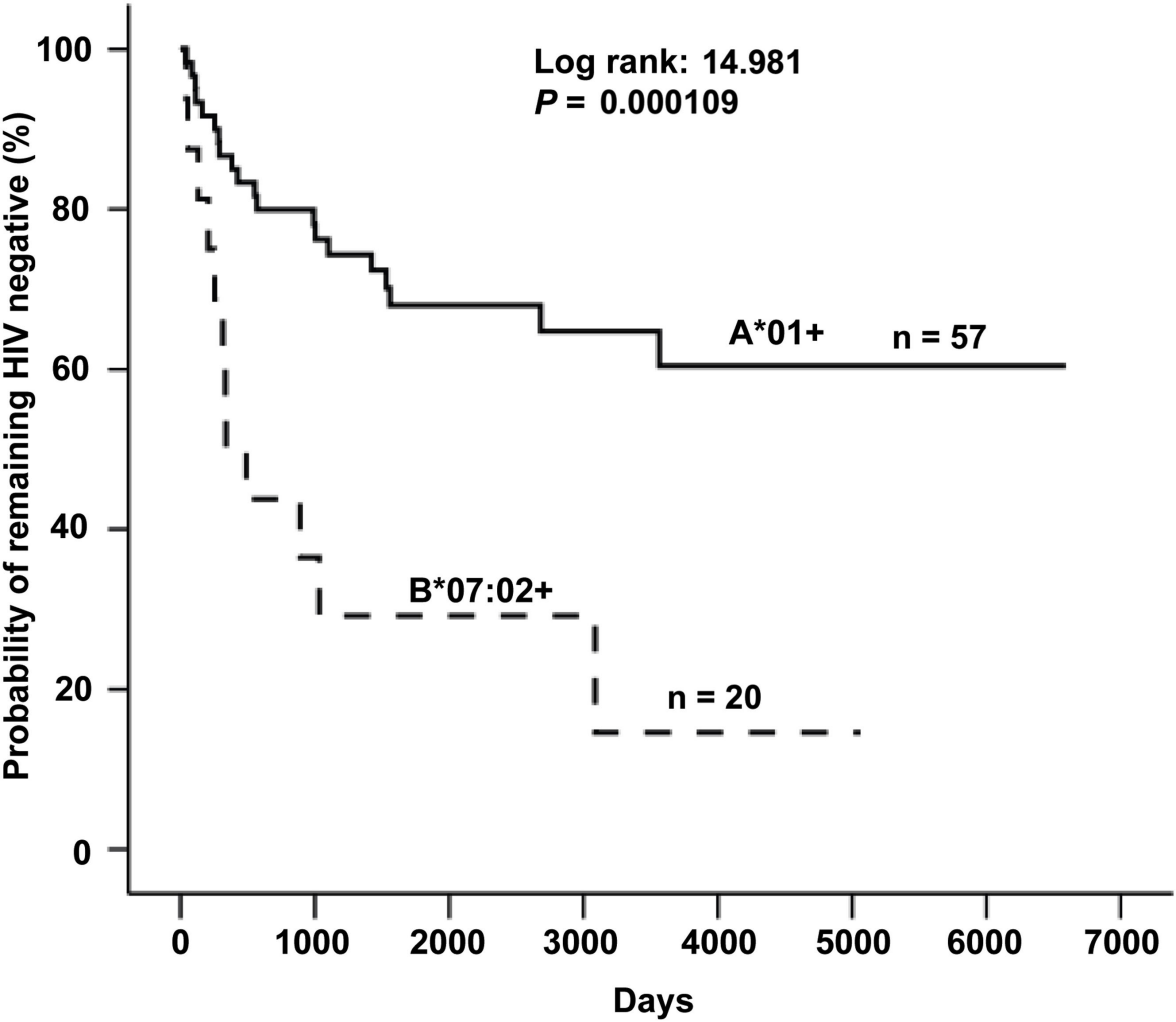
Kaplan-Meier survival analysis comparing HIV seroconversion of A*01+ and B*07:02+ female sex workers enrolled before 2002 in the Pumwani sex worker cohort. The probability of remaining HIV negative for initially seronegative B*07:02+ women (n=20) was significantly lower (p= 0.000109) as compared to women with A*01+ (n=57). Reproduced from Luo M. Natural Immunity against HIV-1: Progression of Understanding after Association Studies. *Viruses*. 2022;14(6):1243, distributed under the terms of the Creative Commons Attribution (CC BY 4.0) license.

The frequency of A*01:01 and B*07:02 in the cohort is >5%, with the A1 supertype reaching 20.5%, and the B7 super type reaching 30.3% in this population (14). Several HLA class I alleles have previously been identified as independently associated with differential rates of HIV-1 seroconversion within the Pumwani cohort. To further characterize the divergent clinical outcomes associated with A*01:01 and B*07:02, Kaplan–Meier survival analysis was performed using data from 328 women who were HIV-1 seronegative at enrolment and entered the cohort prior to 2002, thereby minimizing confounding effects related to the introduction of antiretroviral therapy in 2003. Within this group, individuals expressing B*07:02 (n = 20) exhibited significantly more rapid seroconversion compared to those carrying A*01:01 (n = 57) (**Figure 1**). Consistent with prior observations, HLA-B*07:02–positive sex workers demonstrated a 4.8-fold increased risk of HIV-1 infection relative to HLA-A01:01–positive individuals (p < 0.001). Additional characterization of these Env-specific CD8^+^ T cell epitopes and the T cell responses they elicit is the focus of a companion study currently in preparation.

We determined the time under observation between allele-defined groups at the time of PBMC sampling used for ELISpot assays. The time from cohort enrolment to sampling was comparable between A01:01 and B07:02 groups with a mean of 1728 and 1431 days, respectively (**Table 1**).

### 3.2 A*01:01 and B*07:02 HIV ENV epitopes comparison

**Figure 2:**
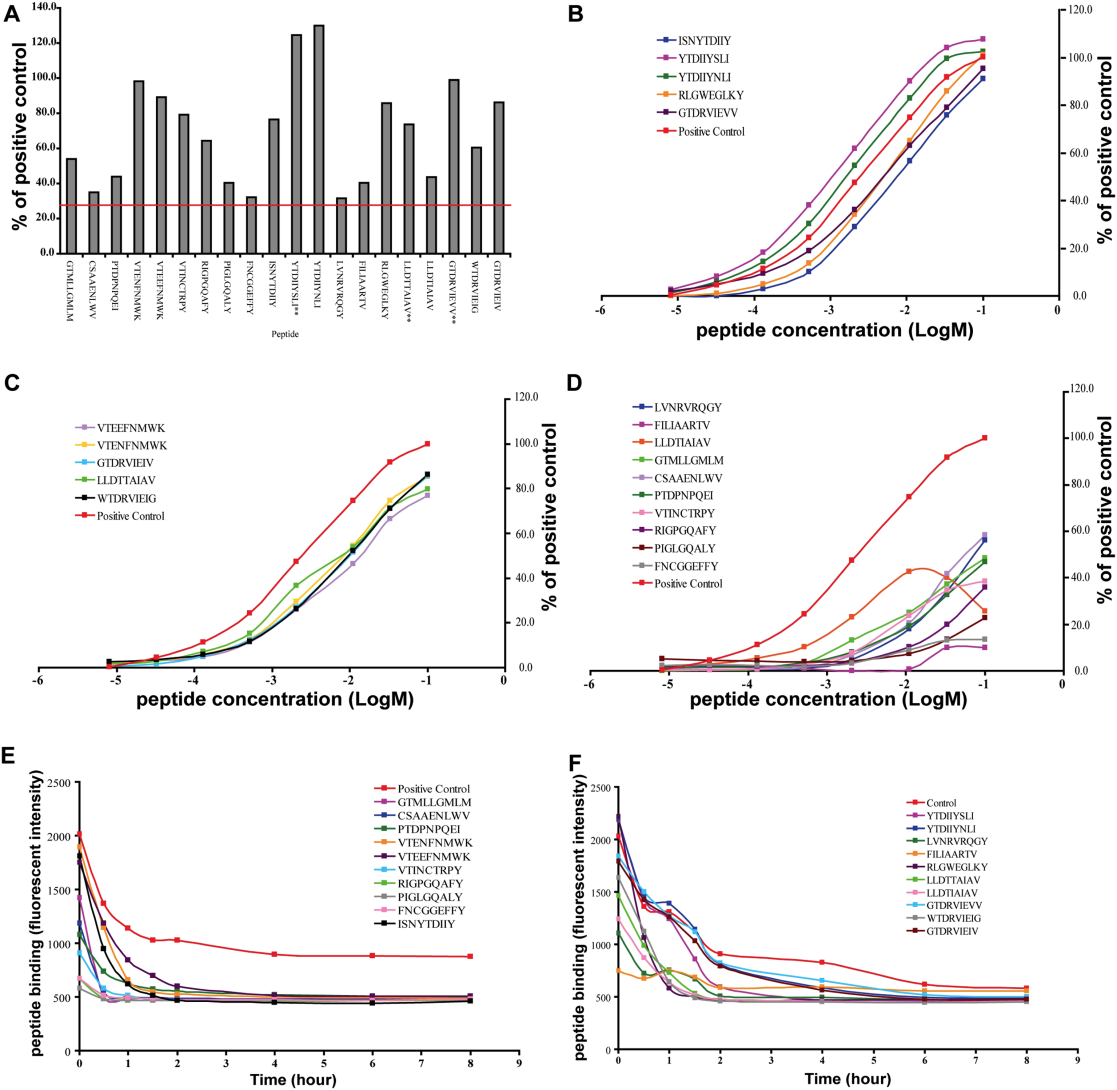
The binding, affinity and off-rate of A*01:01 HIV-1 ENV epitopes and variants. **(A)** The percent binding of ENV epitopes and variants to A*01:01. **(B–D)** The binding affinity of A*01:01 to ENV epitopes and variants. **(E, F)** The off-rates (dissociation rate) of A*01:01 ENV epitopes and variants.

**Figure 3:**
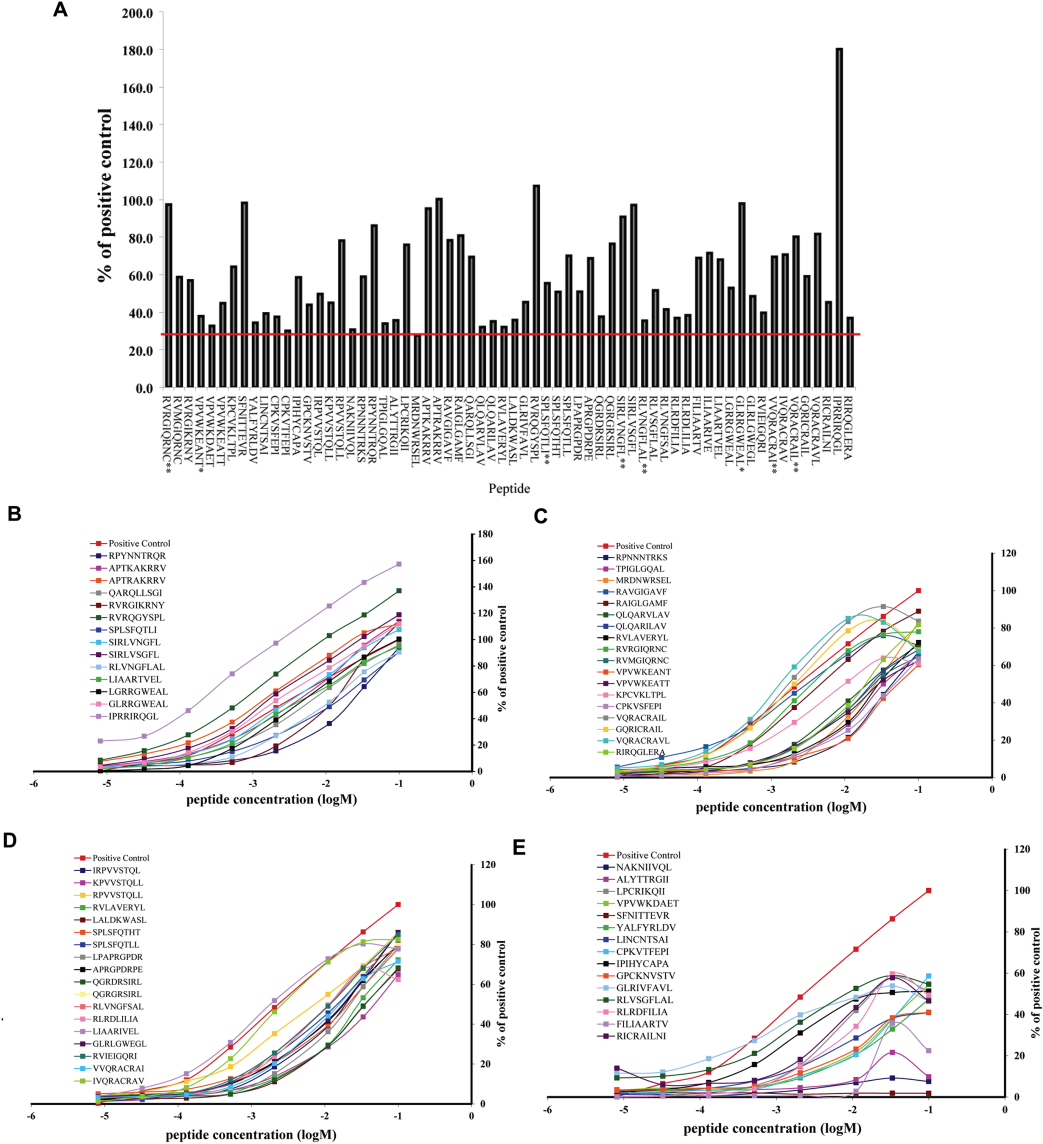
The binding and affinity of B*07:02 HIV-1 ENV epitopes and variants. **(A)** The percent binding of ENV epitopes and variants to B*07:02. **(B–E)** The binding affinity of B*07:02 to ENV epitopes and variants.

**Figure 4:**
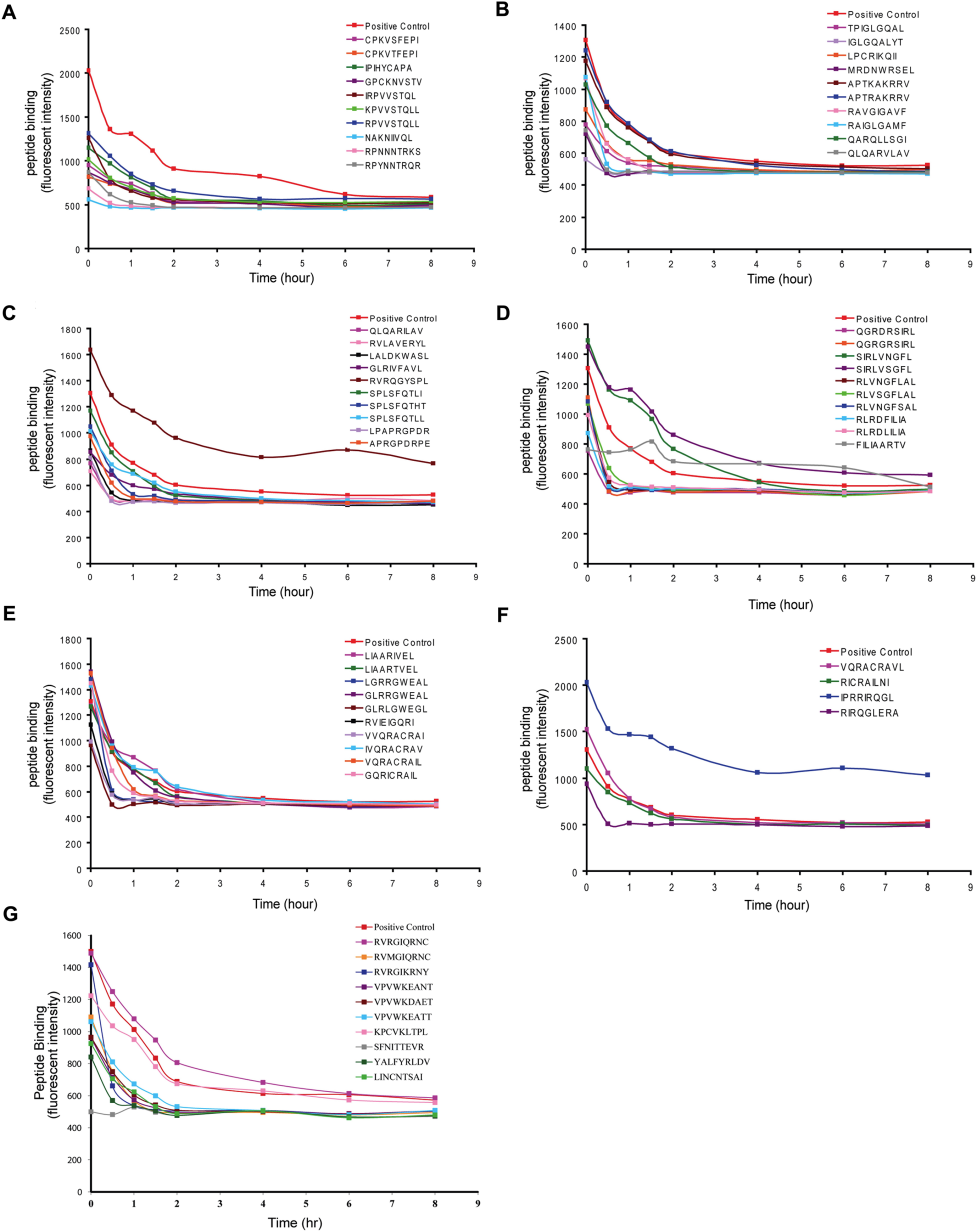
The off-rates of B*07:02 HIV-1 ENV epitopes and variants. **(A–G)** The off-rates (dissociation rate) of B*07:02 ENV epitopes and variants.

Screening of the ENV peptide library using the iTopia Epitope Discovery System identified a subset of peptides capable of binding A*01:01 and B*07:02 above the predefined threshold of 30% relative to the positive control peptide. Among the 1,820 peptides tested, 20 peptides were found to bind *A01:01, corresponding to 14 unique epitopes and 6 sequence variants (**Figure 2**). In contrast, 64 peptides satisfied the binding criteria for B*07:02, comprising 43 distinct epitopes and 21 variants (**Figures 3**, **4****;** **Table 2**). Of these, 15 A*01:01–binding peptides and 43 B*07:02–binding peptides have not previously been reported in the HIV epitope database (**Table 2**).

**Table 2:** Detailed HLA A*01:01 and B*07:02 iTopia binding, affinity and off-rate assay and IFN-γ ELISpot data.

A*01:01 peptide sequence	Location in Env	HIV clade	% peptide binding	Affinity ED50	Off rate t_1/2_ (hrs)	Mean ELISpot response	Reported in HIV database
YTDIIYNLI	GP41	A	129.9	2.73E-06	1.491	131	**N**
YTDIIYSLI*	GP41	A	124.5	2.11E-06	0.958	126	**N**
GTDRVIEVV*	GP41	D	99.0	9.37E-06	1.705	123	**N**
VTENFNMWK*	C1	D	98.2	8.19E-06	0.540	153	Y
VTEEFNMWK	C1	A	89.2	9.96E-06	0.902	160	Y
GTDRVIEIV*	GP41	D	86.2	1.12E-05	1.457	129	**N**
RLGWEGLKY	GP41	A	85.8	7.90E-06	0.390	120	**N**
VTINCTRPY	C2/V3	D	79.2	8.43E-06	0.449	145	**N**
ISNYTDIIY	GP41	A	76.5	8.75E-06	0.449	127	**N**
LLDTTAIAV*	GP41	A	73.7	5.62E-06	0.658	107	**N**
RIGPGQAFY	V3	A	64.3	1.08E-04	1.884	131	**N**
WTDRVIEIG	GP41	A	60.5	1.11E-05	0.529	123	**N**
GTMLLGMLM	Signal	D	54.0	1.75E-05	0.131	434	**N**
PTDPNPQEI	C1	A,D	44.0	3.74E-05	1.321	110	Y
LLDTIAIAV	GP41	A	43.7	1.66E-06	0.680	113	Y
PIGLGQALY*	V3	D	40.4	1.02E-04	3.400	592	**N**
FILIAARTV	GP41	A	40.4	1.90E-05	5.116	135	**N**
CSAAENLWV	Signal/C1	A	35.0	2.45E-05	0.226	102	**N**
FNCGGEFFY	C3	A,D	32.2	8.83E-06	2.234	896	Y
LVNRVRQGY	GP41	D	31.5	5.01E-05	2.124	112	**N**
B*07:02 Peptide Sequence	Env Location	HIV Clade	% Peptide Binding	Affinity ED50	Off Rate t_1/2_ (hrs)	Mean ELISpot Response	Reported in HIV database
IPRRIRQGL	GP41	A,D	180.2	4.98E-07	1.609	114	Y
RVRQGYSPL	GP41	A,D	107.2	1.56E-06	1.519	91	Y
APTRAKRRV*	C5	D	100.2	2.91E-06	1.050	76	**N**
SFNITTEVR	V2	D	98.3	N/A	1.063	284	**N**
GLRRGWEAL*	GP41	D	97.9	4.54E-06	0.821	170	**N**
RVRGIQRNC*	Signal	A	97.3	2.69E-06	1.208	96	**N**
SIRLVSGFL	GP41	A	97.0	3.50E-06	1.251	94	Y
APTKAKRRV	C5	A	95.2	7.09E-06	1.154	123	Y
SIRLVNGFL*	GP41	D	90.8	6.34E-06	0.962	71	**N**
RPYNNTRQR*	V3	D	86.2	3.24E-05	1.096	91	**N**
VQRACRAVL*	GP41	D	81.6	1.06E-06	1.041	132	**N**
RAIGLGAMF*	GP41	D	80.8	5.11E-06	1.166	115	**N**
VQRACRAIL*	GP41	D	80.3	1.80E-06	0.927	99	Y
RAVGIGAVF	GP41	A	78.3	1.72E-06	1.124	72	Y
RPVVSTQLL*	C2	D	78.1	1.74E-05	1.198	149	Y
QGRGRSIRL*	GP41	D	76.4	9.38E-06	1.292	133	**N**
LPCRIKQII	V4/C4	A,D	75.9	5.19E-06	1.300	79	Y
ILIAARIVE	GP41	A,D	71.5	5.95E-06	0.947	87	**N**
IVQRACRAV	GP41	D	70.7	2.29E-06	1.075	118	**N**
SPLSFQTLL*	GP41	D	70.0	2.87E-05	1.013	86	**N**
VVQRACRAI*	GP41	D	69.6	9.02E-06	1.354	86	Y
QARQLLSGI	GP41	A,D	69.4	9.68E-06	1.061	98	**N**
FILIAARTV	GP41	A	68.9	1.32E-05	1.368	84	**N**
APRGPDRPE	GP41	D	68.7	2.63E-05	1.212	73	**N**
LIAARTVEL	GP41	A,D	68.0	1.39E-06	0.981	132	**N**
KPCVKLTPL	C1	A,D	64.2	3.35E-06	1.204	83	Y
GQRICRAIL	GP41	A	59.1	1.47E-06	1.157	127	**N**
RPNNNTRKS	V3	A	59.0	3.72E-05	1.406	98	Y
RVMGIQRNC	Signal	A	58.7	1.17E-05	0.443	88	**N**
IPIHYCAPA	C2	A,D	58.5	1.89E-06	0.904	115	Y
RVRGIKRNY	Signal	D	57.0	1.66E-05	0.278	71	**N**
SPLSFQTLI*	GP41	D	55.4	2.37E-05	0.750	90	**N**
LGRRGWEAL	GP41	D	52.9	6.07E-06	1.137	120	Y
RLVSGFLAL	GP41	A	51.6	1.77E-06	1.233	235	Y
LPAPRGPDR	GP41	D	50.9	2.45E-05	1.671	73	**N**
SPLSFQTHT	GP41	A	50.8	4.53E-05	0.497	103	**N**
IRPVVSTQL	C2	D	49.6	1.99E-05	1.017	96	Y
GLRLGWEGL	GP41	A	48.5	3.33E-05	1.498	86	**N**
GLRIVFAVL	GP41	A,D	45.3	5.57E-07	1.006	100	**N**
RICRAILNI	GP41	A	45.2	4.87E-06	1.100	223	**N**
KPVVSTQLL	C2	A	45.0	9.15E-05	1.192	90	Y
VPVWKEATT*	C1	D	44.8	1.29E-05	0.793	93	Y
GPCKNVSTV	C2	A,D	43.9	1.03E-05	0.862	107	Y
RLVNGFSAL*	GP41	D	41.4	8.29E-06	1.343	103	**N**
RVIEIGQRI	GP41	A	39.7	1.49E-05	1.333	64	**N**
LINCNTSAI	V2/C2	A,D	39.3	6.51E-06	0.635	73	**N**
RLRDLILIA*	GP41	D	38.3	4.79E-06	1.288	105	**N**
VPVWKEANT*	C1	D	37.9	3.76E-05	0.593	78	**N**
QGRDRSIRL	GP41	A	37.7	2.90E-05	1.709	91	**N**
CPKVSFEPI	C2	A	37.5	4.93E-05	0.876	59	Y
RLRDFILIA	GP41	A	36.9	6.53E-06	1.445	102	**N**
RIRQGLERA	GP41	A,D	36.9	1.93E-05	1.411	103	Y
LALDKWASL	GP41	A	35.8	2.57E-05	0.323	78	**N**
ALYTTRGII	V3	D	35.7	5.11E-05	1.845	90	**N**
RLVNGFLAL*	GP41	D	35.5	1.18E-05	1.353	74	**N**
QLQARILAV*	GP41	D	35.1	1.36E-05	1.418	88	Y
YALFYRLDV	V2	A	34.4	4.81E-05	0.506	160	**N**
TPIGLGQAL	V3	D	33.9	2.22E-05	1.334	95	**N**
VPVWKDAET	C1	A	32.7	4.10E-05	0.719	80	**N**
QLQARVLAV	GP41	A	32.0	1.22E-05	1.493	76	**N**
RVLAVERYL	GP41	A	32.0	2.69E-05	1.593	608	**N**
NAKNIIVQL	C2	A	30.6	4.08E-06	1.621	104	**N**
CPKVTFEPI*	C2	D	30.1	9.67E-05	0.916	82	**N**
MRDNWRSEL	C5	A,D	27.4	3.07E-05	1.593	81	**N**

Novel peptides are identified along with their location in the ENV gene. Variants are denoted by a ‘*’.

To determine whether the identified peptides represented true CD8^+^ T cell epitopes, all binding peptides (including sequence variants) were subsequently evaluated using interferon-γ (IFN-γ) ELISpot assays. Peripheral blood mononuclear cells (PBMCs) from A*01:01+ or B*07:02+ women were stimulated with each peptide. All peptides identified as binders by the iTopia system were validated, confirming their capacity to stimulate antigen-specific CD8+ T cell responses (**Table 3**).

**Table 3:** Detailed ELISpot data for HLA A*01:01+ (upper section) and B*07:02+ (lower section) patients tested.

A*01:01 Peptide	Patient #	2462	2468	2646	2426	2297	2326	1938	2261	2178	2672	Responses/ tested
	HIV Status	NEG	POS	POS	POS	POS	POS	NEG	POS	POS	POS	
GTMLLGMLM		20	0	20	0	**155**	**260**	**75**	**95**	**1585**	45	5/10
CSAAENLWV		20	0	20	0	**65**	**205**	**50**	**110**	**125**	**55**	6/10
PTDPNPQEI		5	0	10	0	45	**220**	**85**	**55**	**140**	**50**	5/10
VTENFNMWK		35	15	35	0	**90**	**270**	**120**	**75**	**210**	45	5/10
VTEEFNMWK		40	0	20	0	40	**275**	**160**	45	**155**	**50**	4/10
VTINCTRPY		**75**	0	35	0	**75**	**430**	**60**	**80**	**190**	**105**	7/10
RIGPGQAFY		0	0	45	0	**55**	**300**	45	**100**	**145**	**55**	5/10
PIGLGQALY		10	0	**75**	0	25	**325**	**55**	**70**	**2950**	**75**	6/10
FNCGGEFFY		30	5	**50**	15	30	**265**	**65**	**120**	**4820**	**55**	6/10
ISNYTDIIY		40	5	35	0	**80**	**395**	**80**	**60**	**95**	**50**	6/10
YTDIIYSLI		10	25	**50**	0	10	**270**	45	**95**	**160**	**55**	5/10
YTDIIYNLI		25	25	20	0	**80**	**255**	**135**	**100**	**155**	**60**	6/10
LVNRVRQGY		**55**	0	40	0	**65**	**250**	**95**	**70**	**175**	**75**	7/10
FILIAARTV		0	10	40	0	**60**	**310**	20	**75**	**165**	**65**	5/10
RLGWEGLKY		5	0	45	0	35	**175**	**75**	**90**	**195**	**65**	5/10
LLDTTAIAV		10	10	**55**	15	20	**265**	**50**	**75**	**135**	**60**	6/10
LLDTIAIAV		0	20	45	0	**65**	**285**	**50**	**80**	**140**	**55**	6/10
GTDRVIEVV		20	0	**85**	0	**75**	**305**	**60**	**100**	**160**	**75**	7/10
WTDRVIEIG		0	0	15	0	**95**	**275**	**70**	**60**	**170**	**65**	6/10
GTDRVIEIV		20	0	**80**	0	**125**	**265**	**70**	**90**	**145**	35	6/10
B*07:02 Peptide	Patient #	2619	2423	2425	2231	2644	2337	2427	2320	2400	2545	Responses/ tested
	HIV Status	POS	NEG	NEG	NEG	NEG	NEG	POS	NEG	POS	POS	
RVRGIQRNC		0	**75**	20	0	**165**	0	**80**	**50**	**110**	**95**	6/10
RVMGIQRNC		0	**80**	35	30	**120**	45	**65**	**85**	40	40	4/10
RVRGIKRNY		10	**90**	**75**	20	**55**	0	45	**75**	40	**60**	5/10
VPVWKEANT		0	**95**	15	40	**60**	0	**85**	**50**	**90**	**85**	6/10
VPVWKDAET		5	**95**	10	20	**70**	20	45	35	**85**	**70**	4/10
VPVWKEATT		0	**140**	30	15	**85**	15	35	45	**80**	**65**	4/10
KPCVKLTPL		0	0	40	20	**70**	5	45	15	45	**95**	2/10
SFNITTEVR		0	**230**	0	**60**	**105**	**60**	**50**	35	**1425**	**60**	7/10
YALFYRLDV		0	**415**	15	20	**220**	10	**65**	**55**	**95**	**110**	6/10
LINCNTSAI		0	40	10	10	**110**	40	**55**	**55**	**90**	**55**	5/10
CPKVSFEPI		0	**55**	**65**	5	40	0	20	20	**55**	**60**	4/10
CPKVTFEPI		0	**75**	20	15	**100**	0	**70**	40	**85**	**80**	5/10
IPIHYCAPA		0	25	0	10	**135**	25	**60**	40	**195**	**70**	4/10
GPCKNVSTV		0	**140**	**55**	0	**165**	45	**70**	**60**	**120**	**140**	7/10
IRPVVSTQL		0	30	15	25	**165**	5	**70**	40	**70**	**80**	4/10
KPVVSTQLL		5	**150**	5	15	**135**	35	**60**	**50**	**80**	**65**	6/10
RPVVSTQLL		0	20	25	45	**235**	10	**110**	45	**190**	**60**	4/10
NAKNIIVQL		0	**115**	0	20	**115**	**90**	45	**55**	**145**	35	5/10
RPNNNTRKS		5	0	0	10	**65**	30	**190**	**50**	**115**	**70**	5/10
RPYNNTRQR		0	**65**	0	5	**70**	10	**110**	45	**115**	**95**	5/10
TPIGLGQAL		0	**55**	45	**50**	**110**	10	**85**	**65**	**230**	**70**	7/10
ALYTTRGII		15	**105**	0	15	**135**	0	**50**	**60**	**130**	**60**	6/10
LPCRIKQII		0	0	0	15	**70**	20	35	**60**	**100**	**85**	4/10
MRDNWRSEL		0	**55**	35	15	**110**	0	**50**	35	**120**	**70**	5/10
APTKAKRRV		0	25	25	30	**90**	40	45	**115**	**190**	**120**	4/10
APTRAKRRV		0	25	45	0	**70**	**60**	40	45	**110**	**65**	4/10
RAVGIGAVF		0	0	**55**	20	**50**	40	40	**80**	**100**	**75**	5/10
RAIGLGAMF		0	0	20	20	**135**	30	35	45	**95**	35	2/10
QARQLLSGI		0	0	10	25	**70**	**70**	45	35	**190**	**60**	4/10
QLQARVLAV		0	**70**	25	25	**80**	40	35	30	**85**	**70**	4/10
QLQARILAV		0	25	0	15	**100**	0	35	**60**	30	**105**	3/10
RVLAVERYL		0	**950**	0	**60**	40	25	**1370**	40	**50**	40	4/10
LALDKWASL		0	25	0	15	**55**	**50**	**80**	**60**	**135**	**85**	6/10
GLRIVFAVL		0	**75**	0	0	**125**	45	**95**	35	**110**	**95**	5/10
RVRQGYSPL		0	**80**	5	30	25	5	**70**	**65**	**135**	**105**	5/10
SPLSFQTLI		0	**65**	0	40	**55**	20	**80**	**80**	**145**	**115**	6/10
SPLSFQTHT		0	45	25	5	**95**	**85**	40	40	**125**	**105**	4/10
SPLSFQTLL		0	**85**	45	25	25	20	45	**90**	**75**	**95**	4/10
LPAPRGPDR		0	40	0	20	30	0	**65**	**70**	**75**	**80**	4/10
APRGPDRPE		0	**75**	30	**70**	**85**	40	30	**50**	**90**	**65**	6/10
QGRDRSIRL		0	40	45	0	**120**	**55**	**55**	40	**100**	**125**	5/10
QGRGRSIRL		0	45	10	10	**170**	25	**260**	**70**	**90**	**75**	5/10
SIRLVNGFL		0	0	**55**	0	**50**	5	**50**	**75**	**95**	**100**	6/10
SIRLVSGFL		0	0	**55**	0	**125**	25	**65**	30	**130**	**95**	5/10
RLVNGFLAL		10	5	10	0	**70**	**50**	**55**	40	**100**	**95**	5/10
RLVSGFLAL		0	**75**	25	0	**110**	**95**	25	25	**810**	**85**	5/10
RLVNGFSAL		0	40	30	25	**135**	0	40	35	**110**	**65**	3/10
RLRDFILIA		10	**215**	25	0	**120**	**50**	45	35	**50**	**75**	5/10
RLRDLILIA		5	**95**	0	5	**185**	20	**105**	**55**	**115**	**75**	6/10
FILIAARTV		5	**85**	35	25	**170**	25	40	**55**	**55**	**55**	5/10
ILIAARIVE		0	**55**	10	10	**155**	20	**65**	**85**	**55**	**105**	6/10
LIAARTVEL		20	**195**	0	5	**170**	**70**	40	30	**130**	**95**	5/10
LGRRGWEAL		5	**85**	30	5	**180**	0	40	35	**120**	**95**	4/10
GLRRGWEAL		10	**125**	0	20	**175**	15	45	40	**305**	**75**	4/10
GLRLGWEGL		5	35	0	0	**135**	0	**50**	45	**70**	**90**	4/10
RVIEIGQRI		10	**50**	15	**50**	**115**	35	35	**50**	**60**	**60**	6/10
VVQRACRAI		0	**50**	**75**	0	**195**	0	**50**	**55**	**90**	**90**	7/10
IVQRACRAV		0	0	25	0	**125**	0	35	**85**	**90**	**170**	4/10
VQRACRAIL		5	**75**	0	15	**170**	0	**55**	**75**	**95**	**125**	6/10
GQRICRAIL		15	**105**	30	35	**330**	15	**70**	35	**65**	**65**	5/10
VQRACRAVL		0	0	0	0	**195**	25	**105**	40	35	**95**	3/10
RICRAILNI		0	**200**	0	30	**265**	35	**440**	45	**90**	**120**	5/10
IPRRIRQGL		0	**95**	20	20	**195**	40	**85**	**60**	**105**	**145**	6/10
RIRQGLERA		5	**125**	25	0	**160**	35	45	**65**	**75**	**90**	5/10

POS, HIV-1 positive; NEG, HIV-1 negative at the time of PBMC sampling. HIV status was determined using serologic testing and PCR.

In Bold: ELISPOT Responses greater than or equal to 50 SFU/million.

**Figure 5:**
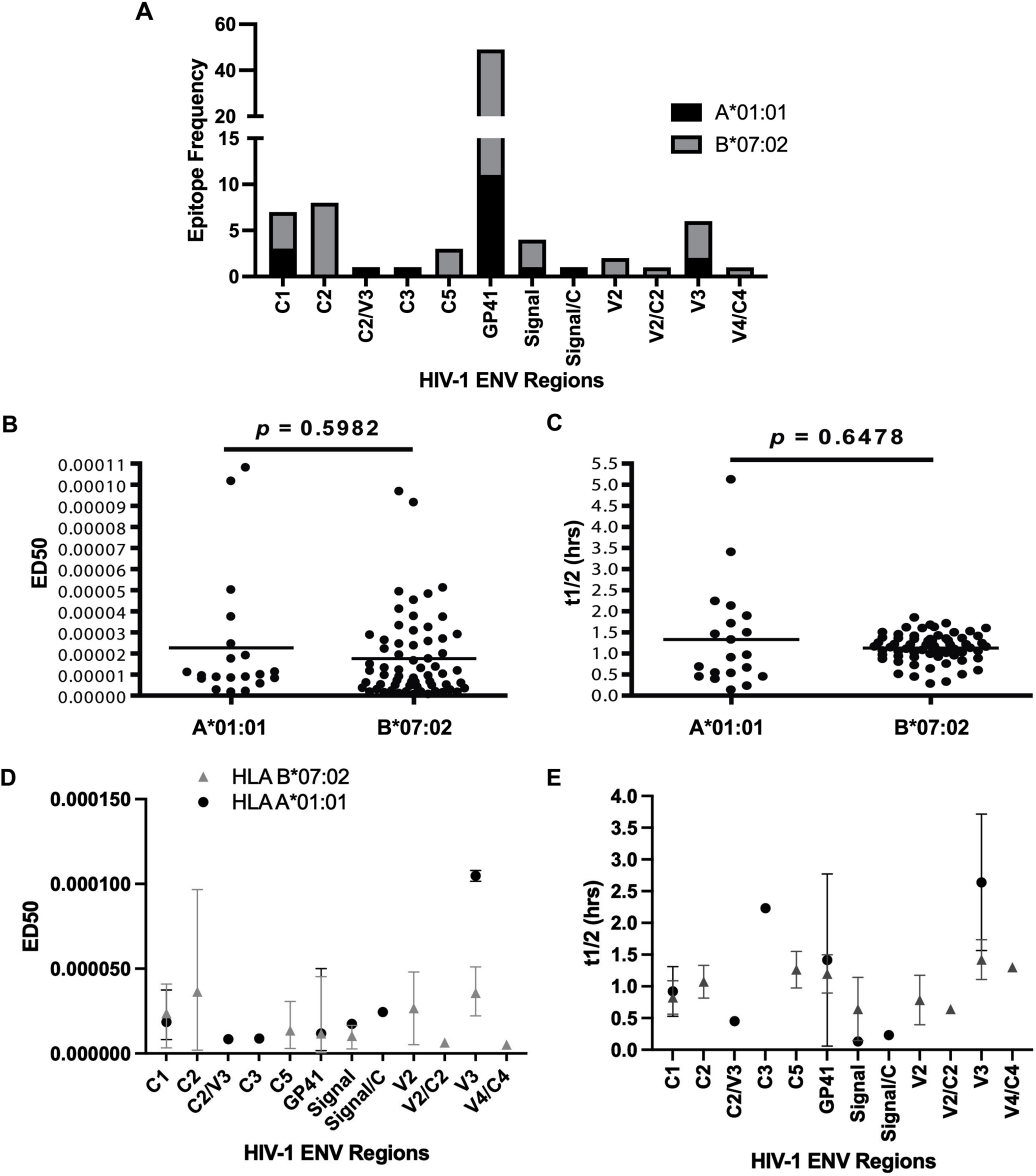
Comparison of the frequency, binding affinity and off-rates of HIV-1 ENV epitopes of A*01:01 and B*07:02. **(A)** The frequency of HIV-1 ENV epitopes and epitope variants of A*01:01 and B*07:02 by ENV region. **(B)** The Binding affinity of HIV-1 ENV epitopes and epitope variants of A*01:01 and B*07:02. **(C)** The off-rates of HIV-1 ENV epitopes and epitope variants of A*01:01 and B*07:02. **(D)** The Binding affinity of HIV-1 ENV epitopes and epitope variants of A*01:01 and B*07:02 as characterized by ENV regions. **(E)** The off-rates of HIV-1 ENV epitopes and epitope variants of A*01:01 and B*07:02 as characterized by ENV regions. Binding affinity and off-rates means and ranges of HIV-1 ENV regions are depicted as lines with either a circle or triangle for A*01:01 or B*07:02, respectively.

The number of ENV epitopes restricted by B*07:02 exceeded those restricted by A*01:01 by approximately threefold. For both alleles, the majority of binding peptides were localized within the gp41 region of ENV, which accounted for 11 of 20 A*01:01–binding peptides and 38 of 64 B*07:02–binding peptides (**Figure 5**). No significant differences were found when comparing the binding affinity and off-rates between alleles (affinity p = 0.5982; off-rate p = 0.6478) (**Figure 5B**), nor were significant differences observed within ENV regions (**Figure 5D**).

Across both alleles, epitopes were more frequently derived from conserved ENV regions (C1–C5) than from variable regions (V1–V5). A*01:01–restricted peptides included only two epitopes or variants within the V3 region, compared to four identified in the C1 and C3 regions. Similarly, B*07:02 recognized six epitopes/variants within the V2 and V3 regions, whereas 15 epitopes or variants were localized within the C1, C2, C4, or C5 regions. Consistent with this distribution, the ratio of conserved to variable region epitopes was calculated as 3.3:1 for A*01:01 and 2:1 for B*07:02 (**Table 2**).

### 3.3 Epitope mapping to HIV-1 clade A/D sequence consensus

#### 3.3.1 Trends in epitope location by HIV ENV region and overlap with CD4-binding sites

**Figure 6:**
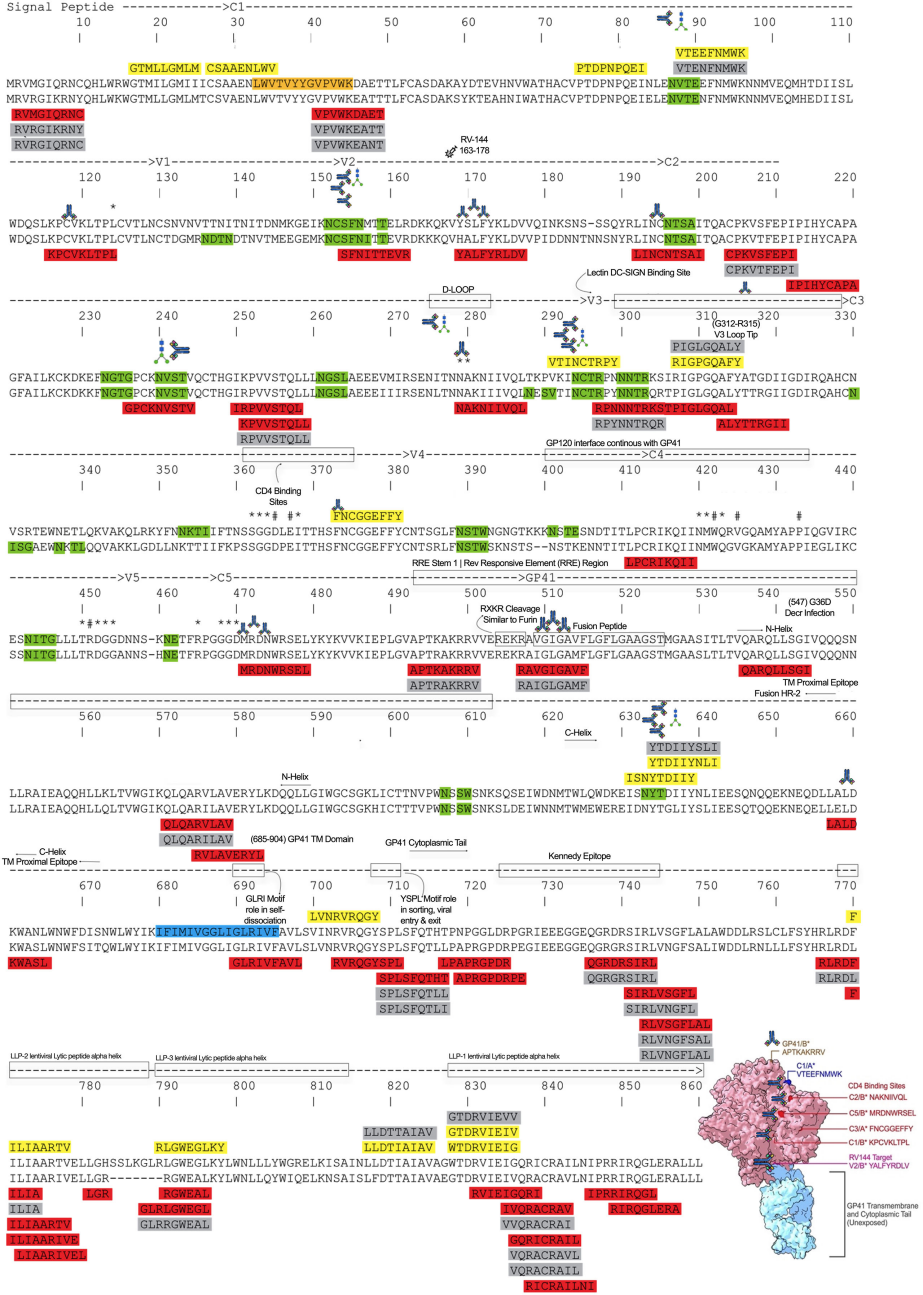
The distribution of A*01:01 and B*07:02 epitopes and variants along HIV-1 Clade A and D. ENV consensus sequence. A*01:01 epitopes are highlighted in yellow and B*07:02 epitopes are highlighted in red. The epitope variants are shaded in grey. The clade A ENV consensus is at the top of the alignment and the clade D consensus is at the bottom. Yellow highlighted, A*01:01 epitopes; Red highlighted, B*07:02 epitopes; Grey shaded, epitope variants; green shaded, N-linked glycosylation site regions; Orange shaded, 4E10 Nab epitope; Blue shaded, transmembrane domain; ^#^the critical CD4-binding sites; *CD4-binding sites; antibody icons, broadly neutralizing antibody targets; vaccine icon, RV144 epitope. Inset, 3D structure of HIV-1 Clade A trimer consensus (PDB:6PWU) with exposed ENV epitopes (ChimeraX v.2025-05-21).

Alignment of A*01:01- and B*07:02-restricted CD8^+^ T cell epitopes with the HIV-1 clade A/D consensus ENV sequence revealed that epitopes from both alleles clustered within three principal regions of the envelope protein: (i) the V3 CD4 co-receptor binding region, including the V3 loop apex; (ii) the gp41 transmembrane domain and cytoplasmic tail; and (iii) the gp41 lentiviral lytic peptide α-helical regions (**Figure 6**). B*07:02-restricted epitopes NAKNIIVQL, and MRDNWRSEL overlapped CD4-binding sites at 2 different locations, while KPCVKLTPL had a single amino acid overlap with a CD4-binding site. One A*01:01 epitope (FNCGEFFY) had such an overlap. Overall, B*07:02-restricted epitopes overlapped CD4-binding sites at a 3:1 ratio relative to A*01:01-restricted epitopes, mirroring the relative abundance of B*07:02 to A*01:01 epitopes identified in this study.

To evaluate the spatial context of these overlapping regions, epitopes were mapped onto a cryo-EM structure of the native HIV-1 Env trimer. This structural analysis indicated that CD4-binding sites containing overlapping CD8^+^ T cell epitopes as well as several additional epitopes identified here, including a RV-144 vaccine-associated B*07:02-restricted epitope YALFYRLDV (55–57) were oriented toward the exterior surface of the Env trimer (**Figure 6**, inset). It should be noted that the cryo-EM model used for this analysis did not incorporate the glycan shield.

#### 3.3.2 The epitopes of A*01:01 and B*07:02 overlap with targets of broadly neutralizing antibodies

**Tables 4** and **5** show the compiled bNAb descriptions from the literature, their epitope characteristics and HIV-1 ENV positions. Mapping the 20 epitopes and epitope variants of A*01:01 discovered in this study, with epitopes of bNAb to the HIV-1 Clade A/D sequence consensus showed that 7 of 20 (~35%) overlapped with the known bNAb-binding sites (**Table 6**; **Figure 6**). All but one of these epitopes overlapped bNAb epitopes that involve glycans, and all overlap sites were within conserved ENV regions. Conversely, mapping the 64 epitopes and epitope variants of B*07:02 and bNAb epitopes showed that 8 of 64 (12.5%) B*07:02 epitopes overlapped known bNAb-binding sites. This number rose to 11 of 64 or (~17%) when including bNAb epitopes that involve glycans, 9 of which were within conserved ENV regions. Thus, a greater proportion of A*01:01 epitopes overlapped with known bNAb epitopes than B*07:02 epitopes with a ratio of nearly 2:1. The ratio of A*01:01 to B*07:02 epitopes that overlapped HIV-1 ENV bNAb epitopes involving glycan sites is 3.14:1.

**Table 4:** Detailed broadly neutralizing antibody (bNAb) descriptions and HIV-1 epitope target regions.

Antibody	Year	Epitope target (env region)	Neutralization breadth (% of strains)	Discovery method	Clades neutralized	Validated	Isolation/comments
b12	1994	CD4 binding site (gp120)	~40% (41% of 190 global isolates; ~75% of Clade B)	Phage display library (asymptomatic donor)	Cross-clade (strongest on clade B; weaker on A, C)	Yes	First bNAb to CD4bs; derived from bone marrow of an HIV+ long-term non-progressor
HJ16	2010	CD4bs-proximal epitope (gp120)	~40% (comparable to b12 on Tier-2 viruses)	Memory B-cell immortalization (EBV)	Cross-clade	Yes	Distinct from b12 epitope
Y498	2017	CD4 binding loop region (C3/C4/C5 on gp120)	~30% (neutralized 30% of 70 isolates)	Phage display library (from CRF07_BC-infected donor)	Cross-clade (tested strains from multiple subtypes)	Yes	Blocks sCD4–gp120 binding
VRC01	2010	CD4 binding site (gp120)	~90% (neutralizes ~91% of strains)	Single B-cell sorting (from donor with broad serum)	Cross-clade (broadly neutralizes A, B, C, etc.)	Yes	Prototype VRC01-class bNAb; crystallized with gp120.
PGT121	2011	V3 glycan supersite (gp120 base)	~60% (neutralized ~58% of panel viruses)	B-cell culture & screening (IAVI Protocol G donor)	Cross-clade (A, B, C; glycan-dependent)	Yes	Potent against many Tier-2 viruses; recognizes N301/N332 high-mannose patch.
2G12	1996	binds 2–4 glycans on gp120 outer domain	~40–50% (≈40% of isolates susceptible)	Hybridoma from HIV^+ donor (human mAb)	Mostly clade B (epitope often absent in clade C)	Yes	Unusual domain-swapped Ab; showed protection in SHIV challenge as dimeric form
2F5	1993	MPER (gp41 membrane-proximal external region). Binds ELDKWA	~60% (neutralized ~60% of 162-virus panel)	Hybridoma (from HIV^+ donor serum)	Cross-clade (except some clade C)	Yes	some autoreactivity noted.
4E10	2003	MPER (gp41, slightly C-terminal to 2F5)	~98% (neutralized 159 of 162 isolates)	Hybridoma (from HIV+ donor serum)	Cross-clade (near-pan neutralization)	Yes	One of broadest bNAbs (targeting MPER helix); binds very conserved epitope; partial autoreactivity.
VRC34.01	2016	Fusion peptide (gp41 N-terminus). 13-residue CDR H3 required for binding.	~50% (50.5% breadth on test panel)	Single B-cell sorting (chronic infection donor)	Cross-clade (A, B, C; FP sequence highly conserved)	Yes	First human bNAb against FP epitope; Basis for fusion peptide vaccine designs.
10E8	2012	MPER (gp41, overlapping 4E10 region)	~98% (neutralizes ~98% of strains)	Single B-cell sorting (slow-progressor donor)	Cross-clade (near-pan neutralization)	Yes	Exceptionally potent MPER bNAb; minimal membrane affinity issues compared to 2F5/4E10. Used in clinical trials (engineered variants) for HIV prevention.
CH01–CH04	2011	V2/V3 apex (quaternary glycan-dependent epitope)	~36–46% (neutralized 36–46% of 91 strains)	Single B-cell lineage isolation (African donor CH0219)	Cross-clade (subset also neutralized by PG9)	Yes	Clonal lineage of 4 related bNAbs; target V1V2 apex glycan (e.g. N160) similar to PG9/PG16.
PG9	2009	V1/V2 apex glycan (trimer-specific)	~79% (neutralized 79% of 162 isolates)	Memory B-cell culture (IAVI Protocol G donor)	Cross-clade (A, B, C, CRFs; apex conserved)	Yes	Requires N160 and other V2 glycans; first V2-apex bNAbs discovered (with PG16). More breadth, slightly less potency than PG16.
PG16	2009	V1/V2 apex glycan (trimer-specific)	~73% (neutralized 73% of 162 isolates)	Memory B-cell culture (IAVI Protocol G donor)	Cross-clade (A, B, C, CRFs)	Yes	Somatic variant of PG9 from same donor; exceptionally potent, with narrow angle of approach. Synergistic with CD4bs bNAbs.
PGT128	2011	V3 high-mannose patch (gp120 N332 supersite)	~70% (neutralizes ~70% of global strains)	Single B-cell sorting (IAVI Protocol G donor)	Cross-clade (prefers viruses with N332 glycan present)	Yes	Ultra-potent V3-glycan bNAb; recognizes cluster of glycans with incomplete neutralization if key glycans missing.
N6	2016	CD4 binding site (gp120; VRC01-class)	~98% (neutralized 98% of HIV-1 isolates)	B-cell lineage maturation analysis (from VRC01 donor)	Cross-clade (near-pan, incl. VRC01-resistant strains)	Yes	Evolved broader than VRC01 by avoiding glycan clashes. Promising for therapy/prophylaxis.
MEL1872	2022	CD4 binding site (gp120; bovine antibody)	~66% (breadth against global panel)	Immunization of cow with ENV trimers (then B-cell isolation)	Cross-clade (tested on clade A and B; broad across subtypes)	Yes	Bovine bNAb with ultra-long CDR H3 (57 aa); extremely potent (IC50 0.009 µg/mL). Demonstrates vaccine-induced bNAb in cows.
ACS202	2016	gp120–gp41 interface (trimer-specific; involves gp41 fusion peptide + glycan N88)	~45% (neutralized 45% of 75-virus panel)	Single B-cell sorting (elite neutralizer donor AMC011)	Cross-clade (broadest in clade C and CRF01_AE)	Yes	Novel cleavage-dependent epitope spanning gp41 FP and neighboring gp120 base. Trimer-preferring
PGT151	2014	gp120–gp41 interface (quaternary, cleavage-dependent)	~66% (neutralized ~66% of strains) ^*^	B-cell sorting (multidonor combined analysis)	Cross-clade (A, B, C; often partial neutralization)	Yes	~26% viruses show incomplete neutralization (plateau <80%). Very potent against sensitive viruses.
Z13e1	~2001 (engineered variant 2006)	MPER (gp41, overlaps 2F5/4E10 epitopes)	~10–20%; Z13e1 affinity-enhanced ~35-fold compared to parent Z13	Phage display (from HIV+ donor, same cohort as 4E10/2F5)	Cross-clade (conserved MPER sequence)	Yes	Originally a weakly neutralizing MPER mAb (Z13).

**Table 5:** Detailed broadly neutralizing antibody (bNAb) HIV-1 sequence consensus target positions.

bNAb	Contact amino acid residues HXB2 numbering	N-Linked glycan contacts by glycosylation site	Structural elements/motifs epitope footprint
b12	G367, D368, E370, I371 (gp120 loop D); G471, D474 (gp120 C4 region)	None required; glycan shielding (e.g. at N386) can modulate access	CD4 binding site – includes loop D and bridging sheet region of gp120.
HJ16	N276, N279, N280 (gp120 C2/outer domain near glycan276); residues in gp120 α5 helix (C4/inner-domain junction)	N276 glycan (binding is critically dependent on glycan at 276)	CD4 binding site (glycan-dependent sub-epitope near V5/outer domain)
Y498	G367, D368, E370, I371, V372 (gp120 loop D); L453, L454, R456 (β23 strand of bridging sheet); G471, D474, R476 (β24–α5 connector in C4)	None; glycan shielding by V1/V2 loop can restrict binding)	CD4 binding site – involves loop D, part of bridging sheet (β23), and C4 region (β24/α5 helix) of gp120.
VRC01	Asp368 (gp120 CD4-binding loop) plus neighboring D loop residues; contacts in outer domain (e.g. 427–430 region) and bridging sheet (e.g. 455–459, 471–474)	None (able to accommodate glycan at N276, which forms a “glycan fence” around the CD4bs)	CD4 binding site – loop D and β-strands of the bridging sheet on gp120.
PGT121	G324, D325, I326, R327 (gp120 V3 base GDIR motif)	N332 glycan (critical);	V3 glycan supersite – base of V3 loop (GDIR region) plus high-mannose patch glycan at N332.
2G12	(No defined peptide residues – epitope is a cluster of high-mannose glycans)	N295, N332, N339, N392 (primary mannose cluster); also contacts peripheral glycans (e.g. N386, N448)	“Silent face” outer domain glycan patch
2F5	E662, L663, D664, K665, W666, A667 (gp41 MPER ELDKWA core)	None	gp41 MPER
4E10	W670, N671, W672, F673, D674, I675, T676, N677 (gp41 MPER NWFDIT region, incl. flanking residues)	None (peptide epitope in MPER; no glycan involvement)	gp41 MPER
VRC34.01	A512, V513, G514…L519 (gp41 fusion peptide residues); plus contacts to gp120 N88 and N241 glycans via CDR loops	N88 glycan; N241 glycan (penetrates between these glycans to access fusion peptide)	gp41 Fusion Peptide (FP); involves conserved glycans
10E8	W672, F673, D674, N677, W680, Y681, I682, K683 (gp41 MPER C-terminal segment)	None (epitope is linear MPER peptide, membrane-proximal)	gp41 MPER – extreme C-terminal neutralizing epitope at base of ENV ectodomain (overlaps and extends past 4E10 region)
CH01–CH04	K168, V169, Q170, K171 (gp120 V2 strand C KXVQK motif)	N160 glycan (required; V2 apex N160 PNGS forms core epitope); often also N156 glycan	V1/V2 apex – quaternary trimer apex epitope involving V2 strand C (lysine-rich segment) and N160 glycan at the trimer 3-fold apex.
PG9	K168, K171 (gp120 V2 strand C basic residues)	N160 glycan (essential); N156 glycan (auxiliary in some strains)	V1/V2 apex – trimer apex β-sheet (strand C of V2 loop) plus conserved N160 glycan.
PG16	K168, K171 (V2 strand C)	N160 glycan (essential); N156 glycan (often contributing)	V1/V2 apex – similar apex epitope as PG9 (strand C with N160 glycan).
PGT128	G324, D325, I326, R327 (gp120 V3 base GDIR motif)	N332 glycan and N301 glycan (dual glycan dependency)	V3 glycan supersite – base of V3 loop (GDIR region)
N6	D368 (gp120 CD4-binding loop) plus other conserved CD4bs residues (overlaps VRC01 contacts)	None (not dependent on N276 glycan; glycan fence is tolerated)	CD4 binding site – VRC01-class epitope on gp120 (loop D and bridging sheet regions);
MEL-1872	Not fully defined; likely contacts canonical CD4bs residues (e.g. D368 on gp120)	None reported (targets peptide surface of CD4bs)	CD4 binding site (features an ultralong CDR H3 that may extend the epitope footprint).
ACS202	A512, G514, L518 (gp41 fusion peptide core); plus gp120 E87 (inner domain loop)	N88 glycan (critical for binding; loss of N88 abrogates binding)	gp41 fusion peptide – N-terminal FP (antiparallel β-strand to bNAb) and adjacent gp120 inner-domain surface (around residue 87).
PGT151	A512, I515, T519 (gp41 fusion peptide; extended conformation); also contacts gp41 helix/loop at interface (gp120-gp41 cleavage region) (allosteric quaternary epitope)	N611 glycan; N637 glycan (gp41 glycans on adjacent protomer required)	gp120–gp41 interface – complex quaternary epitope spanning the fusion peptide (gp41 N-terminus) and gp41 HR1/loop region, plus conserved gp41 glycans (N611, N637); only accessible on cleaved, trimeric ENV.
Z13e1	N671, W672, F673, D674, I675, T676, N677 (gp41 MPER segment overlapping 4E10)	None (linear peptide epitope in MPER)	gp41 MPER – two-segment (bipartite) helical epitope with a hinge at D674, overlapping the 4E10 region of MPER.

**Table 6:** A*01:01 and B*07:02 CD8+ T cell epitopes that overlap broadly neutralizing antibody (bNAb) HIV-1 epitopes.

A*01:01	BNAb	ENV Region	Glycan	CD4-bs	Mean ELISpot Response
VTEEFNMWK	VRC34.01	C1	Y	N	153
VTENFNMWK	VRC34.01	C1	Y	N	160
VTINCTRPY	2G12, PGT121	C2	Y	N	145
FNCGGEFFY	Y498	C3	N	Y	896
YTDIIYSLI	VRC34.01, PGT121, ASC202	GP 41	Y	N	126
YTDIIYNLI	VRC34.01, PGT121, ASC202	GP41	Y	N	131
ISNYTDIIY	VRC34.01, PGT121, ASC202	GP41	Y	N	127
B*0702					
KPCVKLTPL	Y498	C1	N	Y	82.5
SFNITTEVR	CH01-CH04, PG9, PG16	V2	Y	N	284
YALFYRLDV	CH01-CH04, PG9, PG16	V2	N	N	160
LINCNTSAI	B12	C2/V2	N	N	73
GPCKNVSTV	VRC34.01	C2	Y	N	107
NAKNIIVQL	HJ16	C2	Y	N	104
MRDNWRSEL	B12, J498, VRC34.01	C5	N	Y	81
RAVGIGAVF	VRC34.01, PGT121, ASC202	GP41	N	N	72
RAIGLGAMF	VRC34.01, PGT121, ASC202	GP41	N	N	115
LALDKWASL	2F5	GP41	N	N	78

All the T cell epitopes overlapping CD4-binding site clusters are also bNAbs targets: NAKNIIVQL by the N276 glycan-dependent CD4-bs-HJ16 (36, 37); MRDNWRSEL by B12 (first bNAb discovered to block CD4 binding) and other CD4-bNAbs (32–35); KPCVKLTPL by trimer dependent Y498 quaternary epitope recognition (38). The newly isolated neutralizing antibody Y498 targeting gp120 sequences overlap with B*07:02 MRDNWRSEL epitope sequences, and A*01:01 epitope overlaps with CD4-bs FNCGEFFY (32, 38).

**Figure 7:**
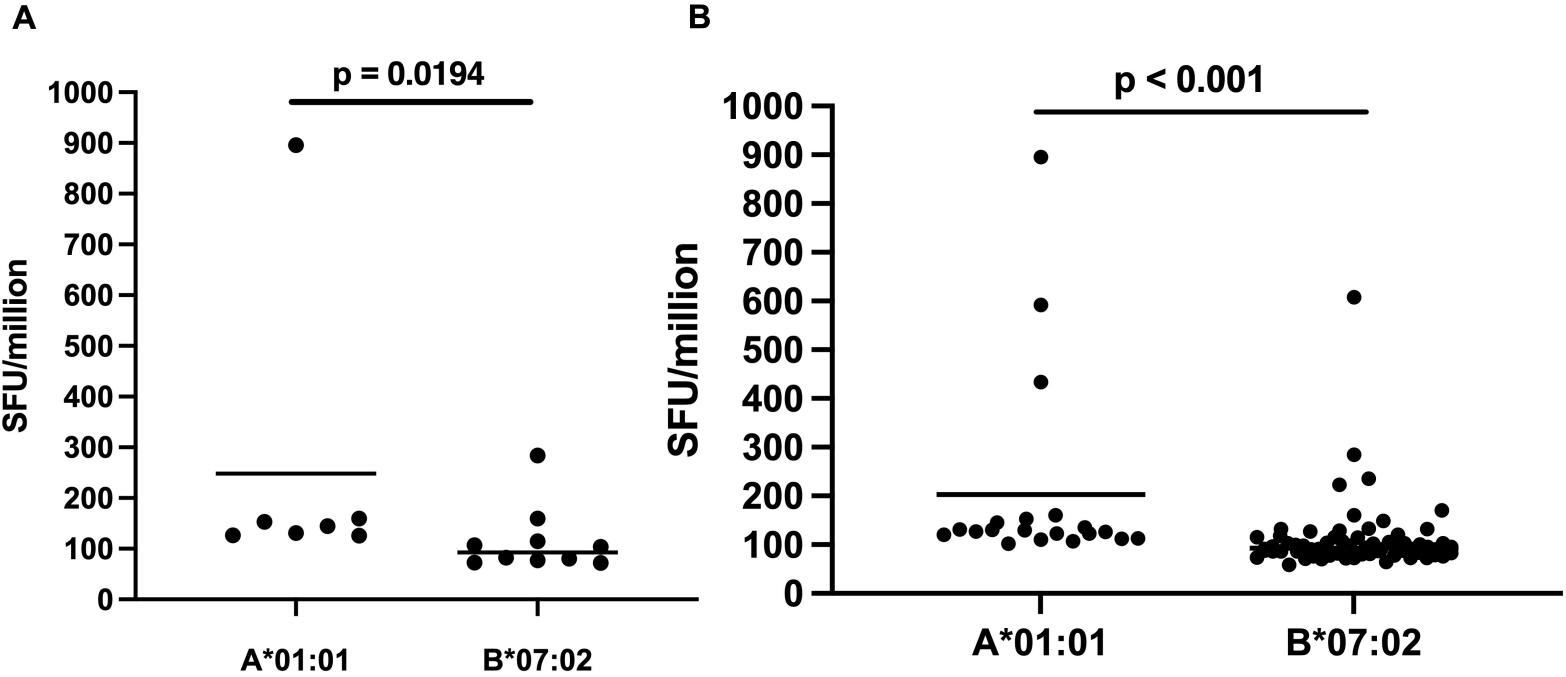
Comparison of the mean IFN-γ ELISpot responses of HIV-1 ENV epitopes and epitope variants of A*01:01 and B*07:02. **(A)** Comparison of the IFN-γ ELISpot responses to the A*01:01 and B*07:02 peptides overlapping epitopes of broadly neutralizing antibodies (bNAb). **(B)** Comparison of the IFN-γ ELISpot responses to the A*01:01 and B*07:02 peptides discovered in this study.

In exploratory analyses of the magnitude of IFN-γ ELISpot responses, bNAb-overlapping A*01:01 peptides showed higher mean SFU compared to bNAb overlapping B*07:02 peptides (**Table 6**; **Figure 7**). The mean SFU of the overlapping CD8-bNAb A*01:01 peptides was 1.22-fold higher than the mean SFU of the A*01:01 epitopes and epitope variants discovered in this study. The mean SFU of the overlapping CD8-bNAb B*07:02 peptides was similar to the overall mean SFU of the B*07:02 epitopes and epitope variants (**Figure 7**). However, the data is influenced by an unequal HIV status distribution of the PBMC donor between alleles. Therefore, these findings are very preliminary, should be interpreted with caution and be tested in future studies. Notably, the potentially surface-exposed A*01:01 peptide FNCGGEFFY, a CD4-bs, and both a CD8+ T cell and bNAb epitope, is the most immunogenic peptide of all those discovered in this study.

## 4 Discussion

### 4.1 Overlap of A*01:01 epitopes as compared to B*07:02 epitopes with bNAb target sites

Both cellular and humoral immune responses play important roles in controlling pathogen infection. A protective HIV vaccine may need to induce coordinated cellular and antibody responses. A*01:01, the allele associated with a 4.8-fold slower HIV seroconversion, recognized 3-fold less HIV ENV peptides than B*07:02, the allele associated with faster HIV seroconversion - similar to the allelic differences in the spectrum of epitopes highlighted in Luo et al. (2012) HIV Gag epitope study (7). In absolute numbers, a slightly lower number of A*01:01-restricted CD8^+^ T cell ENV epitopes overlap known bNAb binding sites than B*07:02 epitopes (7 and 11, respectively). However, a greater proportion of A*01:01 epitopes overlapped known bNAb binding sites than B*07:02 epitopes (ratio of about 2:1). This may imply better coordination between the different arms of adaptive immunity for A*01:01+ individuals, which is linked to A*01:01’s narrow-spectrum CD8+ T cell epitope skew toward conserved ENV regions. We note that contrary to B*07:02, all A*01:01 CD8+ T cell epitopes overlapping bNAb target sites were conserved ENV regions. Amino acid substitutions at any of these functionally critical sites such as the CD4-binding site are likely associated with substantial fitness costs for the virus to evade both cellular and humoral immunity. Although not examined here, it is conceivable that presentation of the conserved ENV epitopes may influence CD4+ T cell-dependent antibody maturation (58, 59). To assess whether CD8^+^ T cell epitopes overlapping known bNAb targets were structurally positioned for potential antibody accessibility, these regions were mapped onto a native prefusion HIV-1 Env trimer cryo-EM model (PDB:6PWU). While this structural model does not incorporate the glycan shield, it represents a conservative, native ENV configuration and demonstrated the plausibility of surface-oriented exposure for these peptides. The model indicates that the potentially surface-exposed A*01:01 epitope FNCGGEFFY, is noteworthy for being the most immunogenic peptide of this study.

### 4.2 Targeting of glycan-shielded regions by CD8+ T cells and antibodies

An intriguing nuance is that many of the CD8+ T cell and bNAb epitope sites involve the virus’s glycan shield. The ENV trimer is heavily glycosylated (N-linked glycans make up ~50% of gp120’s mass) and these glycans normally shield underlying protein epitopes from antibody recognition. However, the majority of known bNAbs can accommodate or even require specific glycans as part of their epitopes. Many potent bNAbs achieve this by using unusually long CDR H3 loops or other structural adaptations to penetrate the glycan shield and contact conserved protein residues beneath it (60–65). The fact that all A*01:01 CD8+ T cell epitopes are within conserved regions denotes functionally critical, structurally constrained regions as explained above. If many such regions happen to be glycosylated, an inference could be that HIV uses glycans to protect exactly those critical and vulnerable regions from bNAbs. Therefore, the disproportionately high A*01:01 CD8+ T cell epitopes and bNAb epitope overlap involving glycans as compared to B*07:02 (ratio of 3.14:1) may also reflect the targeting of structurally constrained regions of ENV. For example, the A*01:01 epitopes YTDIIYSLI, YTDIIYNLI and ISNYTDIIY all lie in GP41’s C-heptad repeat near the membrane-proximal region. This HR2/C-helix is essential for membrane fusion and partly overlaps sites targeted by glycan-dependent bNAbs. Mutations here would impair fusion to the host’s CD4+ cell membrane and potentially make the virus noninfectious.

### 4.3 Study limitations and future directions

This analysis was necessarily limited to broadly neutralizing antibodies that have been identified and characterized to date. As bNAb discovery remains incomplete and biased toward certain clades and infection contexts, additional bNAb targets may exist that are not captured in the current literature. This study did not assess bNAb responses in the study participants themselves. Therefore, the observed overlap reflects convergence at the level of epitope targeting rather than direct evidence of concurrent cellular and humoral responses within the same individual. Functional validation of the observed epitope convergence will be important to determine whether specific HLA class I backgrounds influence viral sensitivity to bNAbs. Future studies incorporating autologous ENV sequences in pseudovirus neutralization assays may help clarify whether HLA genotype is associated with differential susceptibility or resistance to defined bNAb classes, with potential implications for genotype-informed immunotherapeutic strategies. Although this study was restricted to HLA Class I–mediated CD8^+^ T cell targeting, integration of validated HLA Class II–restricted ENV epitopes in future analyses may help clarify the role of CD4^+^ T cell help in the convergence of cellular and humoral immune targeting observed here.

## Data Availability

The original contributions presented in the study are included in the article/supplementary material. Further inquiries can be directed to the corresponding author.
